# Remembering Which X Chromosome to Use

**DOI:** 10.1371/journal.pbio.0020238

**Published:** 2004-07-13

In mammals, males usually have one X and one Y chromosome and females have two X chromosomes. This crucial difference sets the sexes apart, but also creates a problem—female cells have the potential to turn out twice as much X-based gene product as necessary. Males with multiple–X chromosome syndromes face a similar problem. As if to avoid an overdose of X-related proteins, cells in the early embryo inactivate all but one X chromosome. The choice of which X (or Xs) to inactivate is apparently random, but once made, it persists across cell divisions and the specializations that determine a cell's ultimate fate, or type.[Fig pbio-0020238-g001]

**Figure pbio-0020238-g001:**
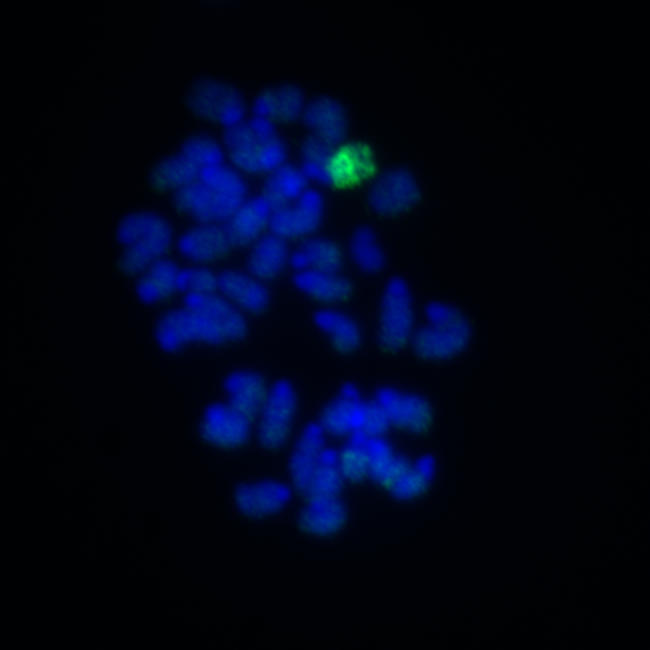
Chromosome-wide histone H3 lysine 27 trimethylation caused by *Xist* expression

A gene called *Xist*—which resides on the X chromosome—has a central role in X chromosome inactivation. It creates a special RNA molecule that spreads from its point of production down the length of the X chromosome, repressing its genes and inactivating the chromosome. After about one cell cycle, this gene silencing no longer requires *Xist* RNA. Daughter cells somehow remember which X to keep mute. In this month's *PLoS Biology*, geneticist Anton Wutz and colleagues at the Research Institute of Molecular Pathology in Vienna show that *Xist* expression during a critical period very early in embryonic development creates a chromosomal memory, independent of X silencing, that might help maintain X inactivation across cell generations.

The molecular underpinnings of X inactivation seem to center on histones, the protein spools around which DNA coils its length. DNA and histones form complexes called chromatin, which undergoes many structural modifications that have important effects on gene expression. For example, tightly packed chromatin inhibits gene expression in its closely curled segments. Not surprisingly, the inactivated X chromosome is coiled into this dense form, called heterochromatin. In the standing model of X inactivation, the *Xist* gene mediates alterations to histones (such as the addition of chemical compounds called methyl groups) along the X chromosome, which result in heterochromatin formation. As this structure is passed on to daughter cells, X silencing is perpetuated.

To explore the molecular changes that mediate X chromosome inactivation, Wutz and colleagues inserted a special *Xist* gene into the X chromosome of male mouse embryonic stem cells, so they could turn *Xist* expression “on” and “off” at will. The stem cells represent the earliest, unspecialized cells of a mouse embryo. Since the cells can be induced to differentiate in culture, they provide the opportunity to study the relationship between differentiation and X chromosome inactivation (which would not normally happen at all in these “male” cells). Using this system, the authors have shown previously that *Xist* must act during a critical window very early during stem cell differentiation—within the first 24 hours. Wutz and colleagues now show that after that window the X chromosome inactivation can still be reversed, but after an additional 24 hours, it cannot. There appears to be a “memory” of *Xist* action, which leads to the permanent shutting down of the chromosome.

The importance of this observation is that it establishes a new step in the process of X chromosome inactivation—between the action of *Xist* and the establishment of irreversible silencing. By looking at the kinetics of histone modification, gene silencing, and *Xist* action, Wutz and colleagues further show that although certain histones are methylated at specific locations during this period in response to *Xist*, these modifications do not themselves constitute the chromosomal memory. The nature of the memory remains mysterious. Further experiments, perhaps looking at different histone modifications, will be required. Clarification of the events that lead to X inactivation will also improve our knowledge of how changes in the organization and structure of chromosomes can influence the activity of genes.

